# Expected clinical competence from midwifery graduates during community service placement in Limpopo province, South Africa

**DOI:** 10.4102/hsag.v23i0.1166

**Published:** 2018-11-29

**Authors:** Khathutshelo G. Netshisaulu, Maria S. Maputle

**Affiliations:** 1 Department of Advanced Nursing Science, University of Venda, South Africa

## Abstract

**Background:**

Community service nurses are placed in a hospital within the first year after qualifying to obtain clinical experience under the supervision of experienced professional nurses. When placed in clinical environments, new midwifery graduates are expected to be job ready, demonstrate competence in the provision of evidence-based care, practise independently and assume accountability and responsibility for their own actions.

**Aim:**

The study aimed at exploring the expectations of experienced midwives of clinical competence of newly graduated midwives during transition.

**Setting:**

The study was conducted at the training hospitals of the five districts in Limpopo province.

**Method:**

The researcher used a qualitative approach which is explorative and descriptive in nature. The population comprised all the professional midwives with experience of 5 years and above working at the selected hospitals in Vhembe, Mopani, Capricorn, Waterberg and Sekhukhune districts of Limpopo province, South Africa. From each selected hospital, five experienced midwives were selected using non-probability, purposive sampling method. An in-depth individual face-to-face interview was used to collect data from the participants, until saturation was reached. The open-coding, Tesch’s eight-step process was used to analyse data.

**Results:**

Results revealed that newly graduated midwives failed to meet the perceived expectations by experienced midwives; this was reflected in sub-themes: limited sense of independence, limited participation in task sharing and commitment and competence versus incompetence to patient care.

**Conclusion:**

The experienced midwives expected newly graduated midwives to function as professionals; unfortunately, they expressed disappointment as graduates did not meet their expectations. Newly graduated midwives lacked sense of independence, commitment to patient care and did not display ability in task sharing.

## Background

The implementation of community service for nurses commenced in January 2008 (Department of Health 2007) and was regulated by the South African Nursing Council (SANC) Regulation 765 of 24 August 2007 (SANC 2010). All nurses completed the 4-year nursing diploma or degree for registration as a nurse (General Community and Psychiatry) and as a midwife to ‘practice a profession in a prescribed category’ and to carry out 1 year of remunerated compulsory community service (Department of Health 2006). When placed in clinical environments, new midwifery graduates are expected to work autonomously and cope with increasingly high acuity patient workloads and major advances in technology (Morrow 2009). According to the SANC, community service must be performed for a period of 12 months. The aim of the community service strategy of the National Department of Health was to retain professional nurses through community service, with graduates obtaining clinical experience under the supervision of experienced professional nurses (Department of Health 2011). During this period, the new graduates must apply their learning from the academic setting into the clinical environment immediately upon entry into practice (Hickey 2009; Meechan, Jones & Valler-Jones 2011).

The hospital managers and experienced midwives expect new midwife graduates to be job ready, demonstrate competence in the provision of evidence-based care, practise independently and assume accountability and responsibility for their own actions (Nursing and Midwifery Board of Australia 2006; Romyn et al. 2009; Wolff, Pesut & Regan 2010a). Although newly licensed midwives have achieved the legal and professional requirements of minimal competence to enter practice, reports have identified stakeholder concerns as to whether graduates are fit for practice (Delaney 2013). Hobbs (2012) reflected a similar view by reporting that many newly qualified midwives lack the clinical skills and judgement needed to provide safe and competent practice. Service managers and experienced midwives continue to have low expectations of the abilities of newly registered midwives to meet the demands of current practice (Avis, Malik & Fraser 2013). The recent review of preregistration midwifery education in the United Kingdom reported that issues over fitness to practise could reflect newly registered practitioners’ lack of confidence in making the transition to a new role (Holland et al. 2010). Delay in the development of confidence amongst new graduates may be related to an unrealistic level of self-expectation or anxiety about how to apply their knowledge when taking on a new role (Avis et al. 2013). According to Skirton et al. (2012), the challenge of transition concerns a change in responsibilities rather than integration into the workplace. Newly qualified graduates were concerned about being professionally accountable for their decisions and actions, which requires confidence as well as competence. They look for organisational support and reassurance as they adapt to the responsibilities of their new role (Avis et al. 2013; Dyess & Sherman 2009).

In the study conducted in Britain, Skirton et al. (2012) made a recommendation for employers to manage transition experiences in order to support and retain their newly qualified workforce. The significance of role transition support was also recognised by Bacon (2010), who reflected on a need for a foundation period of preceptorship for midwifery graduates at the start of their careers to help them make the journey from novice to expert. Skirton et al. (2012) confirmed that the newly graduated midwives were competent to deal with normal childbirth and able to recognise abnormal events appropriately, but the performance was affected as transition from student to a qualified midwife was associated with a drop in confidence; this was more evident when dealing with complex cases and prioritising care when demands were heavy. Avis et al. (2013) reflected a similar version when reporting that newly registered midwives were competent to practise but individual transition journeys require time and structured support to build confidence to deliver quality care to mothers and their babies. As a result, newly graduated midwives were expected to have better time management skills and be more comfortable in the ward environment (Yanhua & Watson 2011). Experienced midwives’ expectations and perceptions towards the new graduates may have a significant impact on the opportunities and experiences available to them during their transition to practice and may therefore affect their performance (Jordan et al. 2013). Schytt and Waldenström (2013) asserted that it is unacceptable to expect undergraduate prepared midwives to have all the necessary skills on qualification because they do not have the additional nursing experience or knowledge. In their opinion piece, they state that structured transition programmes need to be on offer in order to support them safely and effectively into practice (Yanhua & Watson 2011).

Mollart et al. (2011) were of the opinion that experienced midwives in the clinical setting judged all newly graduated midwives as being competent to provide ‘normal’ midwifery care in all areas regardless of qualification. In terms of the graduates’ extended skill base and the ability to manage complications, experienced midwives identified that these would develop over time. Snow (2013) concurred with Mollart et al. (2011) when reporting that the new midwifery graduates were placed as the most experienced midwives on the shift and were expected to take a full clinical load. However, this led to increased anxiety because of fear of making mistakes, lack of knowledge and experience, lack of organisational skills together with the accountability associated with the new role (Rush et al. 2014). Research demonstrates that despite the use of support programmes, midwifery graduates continue to feel underprepared (Evans, Boxer & Sanber 2008; Hillman & Foster 2011). The transition from student to graduate midwife, thus, continues to be stressful and problematic, resulting in increased attrition rates (Clark & Holmes 2007; Milton-Wildey et al. 2014; Newton & McKenna 2007). The main aim of this compulsory placement was to ensure that experienced midwives support, orientate and mentor midwifery graduates in their new role (Govender, Brysiewicz & Bhengu 2017; Zaayman 2016). The positive reports on community service by Roziers, Kyriacos and Ramugondo (2014) state that physicians experienced community service as positive by contributing to their professional development. However, during clinical accompaniment of midwifery students on training, the newly graduated midwives reported their frustrations as they were expected to practise as if they had experience in the clinical areas. On the other hand, experienced midwives reported the lack of commitment and absenteeism on the part of the newly graduated midwives. By identifying areas of concern for new midwifery graduates, nurse educators and academics can potentially re-structure transition programmes and undergraduate curricula to assist graduates in further developing their competence so they are able to deliver safe patient care in their first year of practice and continue to remain in the midwifery workforce.

## Purpose

The purpose of the study was to explore the expectations of experienced midwives regarding clinical competence of midwifery graduates during community service placement. The objective of the study was to describe the expectations of experienced midwives regarding clinical competence of midwifery graduates during community service placement.

## Operational definitions

### Newly graduated midwives

Newly graduated midwives are midwives who have graduated from a 4-year comprehensive nursing programme (either from a university or a college) and are placed at clinical facilities for compulsory community service during their first year of practice.

### Clinical competence

Clinical competence shall refer to the time when newly qualified midwives work competently when providing midwifery care during the transition period.

### Community service placement

Community service is the compulsory service placement at a public facility for a period of 1 year after completion of the 4-year diploma or degree in nursing, before registration as a nurse (general, community and psychiatry) and a midwife.

## Methods

### Design

A qualitative, explorative and descriptive design was used to explore the expectations of experienced midwives of clinical competence from the newly graduated midwives during community service placement.

### Study population

The population comprised all the professional midwives with experience of 5 years and above working at the selected hospitals in Vhembe, Mopani, Capricorn, Waterberg and Sekhukhune districts of Limpopo province, South Africa. From the selected hospitals, 25 participants, 5 from each hospital were sampled using non-probability, purposive sampling method. The experienced midwives supervised the midwifery graduates who have undergone a comprehensive nursing programme (R425 of 19 February 1985, as amended).

### Data collection

Appointments for interviews were secured. Data were collected using unstructured, in-depth, individual, face-to-face interviews, which were conducted in a relaxed conversational manner and each session lasted for 45–60 min. The central question which guided the interview was ‘What clinical competence do you expect from the newly graduated midwives during their transition period when allocated in this unit?’. The question was followed by probing as a communication skill which elicited more information from the participants. The interviews were conducted in English by the researcher under the supervision of the promoter, at the facilities at a convenient time for participants. Interviews were conducted until no new information was emerging with the 23rd participant. Permission to use a voice recorder was obtained and recordings were transcribed verbatim. Field notes were documented during interviews and given meaning.

Data from unstructured interviews were analysed qualitatively using Tesch’s open-coding method (Creswell 2013). The method included the following steps: the researcher read carefully through all the transcripts to get a sense of the whole. After the completion of all transcripts, a list of similar topics was compiled. Data were grouped according to themes and sub-themes and field notes were also coded and categorised. A literature control was done to contextualise the results of the study (Creswell 2013).

## Measures to ensure trustworthiness

Trustworthiness was ensured according to four principles of Lincoln and Guba’s framework, as described in Babbie and Mouton (2005). Credibility was ensured by prolonged engagement, which increased rapport and to clarify descriptions with participants through familiarity. Data triangulation was ensured by using different data collection methods. The researcher collected data through field notes and in-depth individual interviews. Member checking was done to confirm and validate the findings through interviews and discussion with participants in order to discover the truth. For dependability, experts were used to validate the methodology, which was further enhanced by the use of an independent coder to ensure consistency. To ensure conformability, notes were kept safely to enable conduction of an adequate trail and to determine the conclusions, interpretations and recommendations if traced for their sources. Lastly, to ensure transferability, the findings were not transferred to other setting but a dense description of the results was done to make it possible for another person to make comparison if needed.

### Ethical considerations

Ethical clearance to conduct the study was obtained from the University of Venda Research Ethics Committee (SHS/16/PDC/06/1304). Limpopo Provincial Department of Health (Ref 4/2/2) as well as selected hospitals’ managers granted permission to access the facilities. Participants gave written, informed consent and were informed of their right to withdraw from the study without any penalty. Ethical principles of fairness, privacy, confidentiality, anonymity as well as participants’ rights to voluntarily participate in the study were considered.

## Findings

### Presentation of findings

Data were collected from 25 experienced midwives who were working in labour wards of the five selected hospitals in Limpopo province. Data from participants were consolidated and linked to each other to form clusters, a theme and sub-themes emerged as presented in Table 1.

**TABLE 1:** Summary of findings as expectations of experienced midwives.

Theme	Sub-themes
1. Ability to function as professional midwives	1.1. Limited sense of independence 1.2. Task sharing in the unit 1.3. Commitment to and competence versus incompetence in patient care

## Discussion of findings

### Theme: Ability to function as professional midwives

As a caring profession, midwifery is a practical discipline in which students develop complex psychomotor skills, cognitive thinking as well as affective skills which are applied in midwifery clinical setting. The nursing education and midwifery facilities should seek to promote midwifery clinical skills, empowering students with scientific knowledge by deploying teaching strategies, which enhance critical and analytical reasoning abilities (Moeti, Van Niekerk & Van Velden 2004).

Experienced midwives’ expectations from new graduates may impact on the opportunities and experiences available to them during their transition to practice. At the same time, the new graduates’ perceptions of their own level of knowledge, skills and expertise on registration will influence how they approach their transition to practice (Cubit & Ryan 2011). This was supported in the study by Moeti et al. (2004) when pointing that to facilitate the ability to function as a professional midwife, it is important to conduct an orientation programme for the newly qualified midwives to ensure that they receive information, which would help them to function as competently as possible.

Sub-themes that emerged under this theme were limited sense of independence, task sharing in the unit and commitment to and competent versus incompetent patient care

#### Sub-theme 1.1: Limited sense of independence

Results revealed that experienced midwives expected newly graduated midwives to be competent and behave like independent practitioners. When newly graduated midwives failed to behave likewise, experienced midwives became frustrated.

This was confirmed by the following excerpt:

‘I am so disappointed because I thought that the graduates will be able to function as independent professionals, instead they are not. When you are working with them it’s the same as when working with students. They are not fit to work as professional midwives, you always need to be with them at all times and that’s so frustrating.’ (Experienced midwife, participant 3, MR hospital)

Carter et al. (2013) supported the above when they reported that experienced midwives in the clinical setting judged all newly graduated midwives as being competent to provide ‘normal’ midwifery care in all areas regardless of the experience.

This was confirmed by a participant stating:

‘You expect extra hands, instead they need you wholly. There are those who need assistance in simple procedures such as admission of a woman in labour. You ask yourself if such graduates never went to the clinical area during their training. When they realise you are committed to help them, they will make a streamline following you; this makes you not to complete your job as you will be attending to them. In that situation, you feel it would be better if you were alone because they are just a burden to you.’ (Experienced midwife, participant 1, TR hospital)

In their study, Mason and Davies (2013) found that experienced midwives had very high expectations of newly qualified midwives once they were in practice, along with an assumption that qualified meant ‘all knowledgeable’. Dixon et al. (2014) concurred when they highlighted how practising midwives’ expectations of newly qualified midwives were unrealistic, suggesting that pressures of the ward environment, being able to adapt and integrate quickly and the added responsibility of accountability were particularly overwhelming.

A participant said:

‘These graduates don’t want to remain in the unit alone and say if something happens they will be accountable. That is so frustrating because we are banking on them as they are no longer students. We end up changing our off duties in order to remain with them. Some don’t want to go to theatre alone to receive new-born babies. They say they don’t feel confident enough to do that alone.’ (Experienced midwife, participant 3, MP hospital)

This is confirmed by Feltham (2014), who reported that newly graduated midwives do not feel safe to remain with the ward alone. They need time to familiarise themselves with the new work situation and to develop the competence necessary to assume full responsibility (Feltham 2014). Avis et al. (2013) revealed that on receiving qualification, participants realised that the protection and support offered by their preceptors during their training was abruptly withdrawn and such withdrawal made them feel like they were abandoned resulting in clinging to the experienced midwives for support. Jordan et al. (2013) concurred by reporting the assimilation anxiety experienced by newly graduated midwives as they are suddenly expected to assume responsibility for their own patients, together with a loss of sheltered academia, which made them feel vulnerable. Moeti et al. (2004) and Govender et al. (2015) have stated that in the quest for quality care, the nurse manager should attach newly registered midwives to experienced midwives for supervision and further learning. Supervision facilitates the integration of theory and practice and improves the graduates’ ability to provide safe and efficient care.

#### Sub-theme 1.2: Task sharing in the unit

Task sharing refers to the delegation of tasks to the less specialised health workers (WHO 2007; WHO/PEPFAR/UNAIDS 2008). In this context, it would also refer to giving in-service education to the midwifery graduates and then allowing them to take activities they have not undertaken before (WHO 2012). Managing the workload in any area of maternity care is a challenge all midwives face, whether they are newly qualified or not (Mollart et al. 2011). Results revealed that experienced midwives expected newly graduated midwives to play a major role in task sharing of workload; unfortunately the new graduates couldn’t meet their expectations.

‘It’s so frustrating because we thought our workload will be reduced as we are now having extra hands. Instead they become a problem because they can hardly perform any single procedure alone. You should always be there for assistance whenever they are performing duties.’ (Experienced midwife, participant 1, LR hospital)

Another participant stated:

‘Sometimes you feel like it would be better if you were alone because you spend so much time guiding and supervising them as if you are working with a student. We thought our lives were going to be better as we thought they would reduce the workload, instead they make the workload to be doubled.’ (Experienced midwife, participant 3, SRR hospital)

In their study of newly graduated midwives in New Zealand, McCarthy et al. (2013) found that graduates struggle to fit in and are unable to develop the confidence and competence to positively contribute to the workload. This is because they may not have the required level of skills or expertise, leaving the experienced midwives struggling with the workload (McCarthy et al. 2013).

A participant stated:

‘We were so happy when we realised they were going to form part of our staff, because we are over-worked due to shortage of staff. But we are so disappoint because there is no reduction of workload at all. One day I delegated one to go to theatre to receive a new-born baby. The response I got was, “I don’t feel confident enough to go to theatre alone”.’ (Experienced midwife, participant 5, SRR hospital)

This was confirmed by another participant who said:

‘We are short staffed to provide close supervision, and it’s like these graduates do not take that seriously because they still demand your attention even if you are alone.’ (Experienced midwife, participant 4, MR hospital)

Task sharing between midwives and midwifery graduates could lead to equal task division and increase productivity across ones with sufficient workforce (Fakhri & Aryankhesal 2015).

Yanhua and Watson (2011) pointed that healthcare administrators face challenges to get competent and experienced midwives and are forced to find an option such as hiring newly graduated midwives to function independently and fill the shortage of competent and experienced midwives.

#### Sub-theme 1.3: Commitment to and competence versus incompetence in patient care

The 4-year, preregistration route into midwifery was found to be effective in the preparation for midwifery practice as judged against a model of a competent midwife at a point of registration. However, evidence suggests that not all students were equipped to practise competently and confidently in context of uncertainty and change (Panzavecchia & Pearce 2014).

‘Some of the new graduates are committed to provision of care to patients; the problem is that they are not competent. Some are competent but do not have confidence in what they do as a result, performance of procedures becomes very slow.’ (Experienced midwife, participant 2, TR hospital)

In their study, Fenwick et al. (2012) reported that participants are committed to provision of quality care, but the problem is that during transition they still feel less competent and less confident; therefore, they need support from the experienced professionals. Unfortunately, support is not there and that makes them resort to being mischievous as a way out.

One participant said:

‘These graduates are not serious, neither are they committed. If a cell phone rings whilst attending to the patient, he/she stops everything and attends to a cell phone. They are not committed; you cannot even risk leaving them running a shift.’ (Experienced midwife, participant 1, LR hospital)

This was supported by another participant from TR who said:

‘We are so shocked because we thought they will be committed to render quality care to patients, but they are not. Some openly verbalize that they are not even interested in working in the labour ward they are just complying with the placement policy.’ (Experienced midwife, participant 3, TR hospital)

Young (2012) revealed that newly graduated midwives find it difficult to make independent decisions and high levels of responsibility and accountability lead to anxiety, which negatively affect their commitment regarding performance of care to patients. Jordan et al. (2013) concurred when they reported that newly qualified midwives demonstrated a very low level of commitment to provision of care. This was because of the increased level of anxiety resulting from fear of making mistakes, lack of knowledge and experience, lack of organisational skills together with the accountability associated with the new role (Jordan et al. 2013).

One participant said:

‘Some graduates first check the duty roster. When they realise they are remaining with the supervisor they don’t like, they absent themselves from duty, faking illness. They don’t even hide it they tell their friends that I won’t come tomorrow because I don’t want to work with so and so.’ (Experienced midwife, participant 4, MP hospital)

Another participant stated:

‘Some of the midwifery graduates have tendency to absent themselves especially if on duty for 8 consecutive days which is strenuous. Therefore they either report sick a day or two before going for resting days or vice versa. Or report sick if their requisitions for special off duties have not been approved.’ (Experienced midwife, participant 3, MR hospital)

Fenwick et al. (2012) indicated that experienced midwives rated some newly qualified midwives as competent regarding the necessary skills and knowledge to perform the roles expected of them, the only problem was lack of interest in midwifery field and this affected their commitment. This was confirmed by Crombag et al. (2013), who assessed newly qualified midwives and concluded that they were fit for practice at the time of their professional registration; the only thing that could not be guaranteed was the level of commitment.

## Recommendations

- A collaborative forum should be established between the nursing education institutions, the hospital management and the nursing education section of the hospital. The aim of the forum should be to facilitate the working together and the understanding on the needs of new graduates during transition. An effective collaboration between the nursing college, the universities and the service could facilitate maximisation of quality and quantity in nursing education for the province.
- A contextual transition programme should be developed and implemented. The programme should be used to orientate, supervise and mentor midwifery graduates before they are expected to function as independent practitioners.
- The collaborative forum should revive the availability of preceptors and mentors who will facilitate the implementation of the transition programme. The programme should focus on orientating and mentoring the new graduates.
- Institutional policies should also be clarified to the graduates so that they become conversant with effective functioning of the institution.
- Task sharing should be strengthened and formalised within the hospital units.

## Conclusion

The study focused on the expectations of experienced midwives during transition of newly graduated midwives. The findings revealed a theme and three sub-themes. It was found that newly graduate midwives did not meet the expectations as they lacked a sense of independence and commitment to patient care and could not perform delegated duties towards ward coverage by experienced midwives, resulting in increased workload and frustration on the part of experienced midwives. Hence, a contextual transition programme should be developed and implemented to orientate, supervise and mentor midwifery graduates before they are expected to function as independent practitioners.
